# 

**Published:** 1880-11

**Authors:** 


*CONTENTS.*

| | Page |
|---|---|
| Ventilation | 203 |
| Consumption | 205 |
| Health and Fun | 212 |
| A Lesson to Parents | 213 |
| Sun Baths | 216 |
| Inhalation | 218 |
| Too White Bread | 220 |
| Retiring from Business | 220 |
| Preventable Society | 224 |
| Simples | 224 |
| Sound Sleep | 225 |
| Threatenings of Disease | 226 |
| The Good Physician | 226 |
| Hydrophobia | 227 |
| Potatoes as Food | 228 |
| Health Recreations | 230 |
| Lifetimes | 230 |
| Onions | 231 |
| Men Wanted | 231 |
| A Glass of Brandy | 232 |
| The Lifting Cure | 233 |

